# The pharmacological profile of ELIC, a prokaryotic GABA-gated receptor

**DOI:** 10.1016/j.neuropharm.2012.05.027

**Published:** 2012-09

**Authors:** Andrew J. Thompson, Mona Alqazzaz, Chris Ulens, Sarah C.R. Lummis

**Affiliations:** a Department of Biochemistry, University of Cambridge, Cambridge CB2 1QW, UK; b Laboratory of Structural Neurobiology, KU Leuven, Leuven, Belgium

**Keywords:** Cys-loop, *Erwinia*, Ligand-gated, Antagonist, Nicotinic, ELIC, GLIC, GABA, nACh, nicotinic acetylcholine, AChBP, acetylcholine binding protein, GABA, γ-aminobutyric acid, ELIC, *Erwinia* ligand-gated ion channel, GLIC, *Gloeobacter* ligand-gated ion channel, 5-AV, 5-aminovaleric acid, GHB, gamma-hydroxybutyric acid, PXN, picrotoxinin, ACh, acetylcholine, 5-HT, 5-hydroxytryptamine

## Abstract

The *Erwinia* ligand-gated ion channel (ELIC) is a bacterial homologue of vertebrate Cys-loop ligand-gated ion channels. It is activated by GABA, and this property, combined with its structural similarity to GABA_A_ and other Cys-loop receptors, makes it potentially an excellent model to probe their structure and function. Here we characterise the pharmacological profile of ELIC, examining the effects of compounds that could activate or inhibit the receptor. We confirm that a range of amino acids and classic GABA_A_ receptor agonists do not elicit responses in ELIC, and we show the receptor can be at least partially activated by 5-aminovaleric acid and γ-hydroxybutyric acid, which are weak agonists. A range of GABA_A_ receptor non-competitive antagonists inhibit GABA-elicited ELIC responses including α-endosulfan (IC_50_ = 17 μM), dieldrin (IC_50_ = 66 μM), and picrotoxinin (IC_50_ = 96 μM) which were the most potent. Docking suggested possible interactions at the 2′ and 6′ pore-lining residues, and mutagenesis of these residues supports this hypothesis for α-endosulfan. A selection of compounds that act at Cys-loop and other receptors also showed some efficacy at blocking ELIC responses, but most were of low potency (IC_50_ > 100 μM). Overall our data show that a number of compounds can inhibit ELIC, but it has limited pharmacological similarity to GLIC and to Cys-loop receptors.

## Highlights

► ELIC is structurally and functionally similar to Cys-loop receptors. ► ELIC can be activated by GABA but not other GABA_A_ receptor agonists. ► ELIC responses are blocked by some compounds that block the channel of GABA-activated and other Cys-loop receptors. ► The potency of the compounds that block ELIC is lower than in Cys-loop receptors and in GLIC. ► ELIC pharmacology is distinct from that of related receptors.

## 1 Introduction

The Cys-loop family of ligand-gated ion channels are membrane proteins responsible for fast excitatory and inhibitory synaptic neurotransmission in the central and peripheral nervous systems. Members of this family share a common quaternary structure of five subunits that can be homomeric or heteromeric. Each of the subunits has three distinct regions that are known as the extracellular, transmembrane and intracellular domains. The N-terminal extracellular domain contains the neurotransmitter binding sites, which are located at subunit interfaces. They are created by the convergence of three amino acid loops (loops A–C) from the principal subunit and three β-sheets (loops D–F) from the adjacent complementary subunit (Brejc et al., 2001; Unwin, 2005). The transmembrane domain consists of 4 transmembrane α-helices from each subunit (M1–M4) that span the membrane, with the M2 helices surrounding the central ion pore. The intracellular domain is largely unstructured, and is responsible for receptor trafficking, regulation by intracellular modulators, and has a role in channel conductance (Hales et al., 2006; Deeb et al., 2007; Carland et al., 2009).

**Fig. 1:**
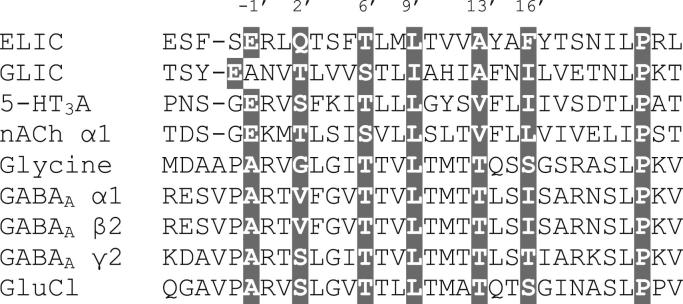
An alignment of channel-lining residues for a range of eukaryotic Cys-loop receptors and prokaryotic homologues. As is common for these receptors, a prime notation is used to facilitate comparison between different subunits, with 0′ being the conserved charged residue at the start of M2. Grey indicates residue conservation. Accession numbers are: ELIC P0C7B7, GLIC Q7NDN8, 5*-*HT_3_P46098, nACh α1 P02708, Gly P23415, GABA_A_ α1 P14867, GABA_A_ β2 P47870, GABA_A_ γ2 P18507, GluCl Q94900.

One of the major problems in understanding the mechanisms of action of this family of channels is the paucity of high resolution structures. Nevertheless the identification of prokaryotic Cys-loop receptor homologues has significantly improved our understanding of many structural details (Tasneem et al., 2005). An X-ray crystal structure of a Cys-loop receptor homologue from *Erwinia chrysanthemi* (*Erwinia* ligand-gated ion channel or ELIC) was solved in 2008, and one from *Gloeobacter violaceous* (*Gloeobacter* ligand-gated ion channel, or GLIC) in 2009 (Hilf and Dutzler, 2008, 2009; Bocquet et al., 2009). These prokaryotic receptors share many of their structural features with Cys-loop receptors, although they do not possess an N-terminal α-helix, an intracellular domain, or the disulphide bonded loop that gives the eukaryotic family its name. The crystallisation conditions of these proteins (ELIC unliganded; GLIC at high pH) led to the proposal that ELIC is in a closed conformation, while GLIC is in an open conformation, although recent work suggests that the structure of GLIC may represent a desensitized state (Parikh et al., 2011). GLIC is activated by protons and ELIC is activated by a range of small amine molecules, including GABA (Ulens et al., 2011; Zimmermann and Dutzler, 2011). The potency of GABA on ELIC is low compared to its eukaryotic counterparts, but work on bacterial receptors in other systems (e.g. Singh et al., 2007; Zhou et al., 2007), suggest that even if the potencies are not in the same range, their mechanism of action at homologous proteins are similar, making ELIC an attractive model system to understand the molecular mechanisms of Cys-loop receptors. Although ELIC shows low sequence similarity with Cys-loop receptors overall, it shows high sequence homology (>60%) in the M2 region (Fig. 1). The pharmacology of ELIC, however, has still not been comprehensively explored. Here we report the effects of a range of compounds that could potentially activate or inhibit the receptor.

## 2 Materials and methods

### 2.1 Cell culture and oocyte Maintenance

*Xenopus laevis* oocyte-positive females were purchased from NASCO (Fort Atkinson, Wisconsin, USA) and maintained according to standard methods. Harvested stage V–VI *Xenopus* oocytes were washed in four changes of ND96 (96 mM NaCl, 2 mM KCl, 1 mM MgCl_2_, 5 mM HEPES, pH 7.5), de-folliculated in 1.5 mg ml^−1^ collagenase Type 1A for approximately 2 h, washed again in four changes of ND96 and stored in ND96 containing 2.5 mM sodium pyruvate, 50 mM gentamycin, 0.7 mM theophylline.

### 2.2 Receptor expression

The ELIC sequence (Genbank accession number POC7B7) was purchased from Genscript as a synthetic gene with optimized codon usage for expression in *Escherichia coli*. For electrophysiological recordings from *Xenopus* oocytes, the mature sequence of ELIC (residue numbers 8-322) was cloned into pGEMHE with the signal sequence (MRCSPGGVWLALAASLLHVSLQ) of the human α7 nACh receptor (Liman et al., 1992). cRNA was *in vitro* transcribed from linearised pGEMHE cDNA template using the mMessage mMachine T7 Transcription kit (Ambion, Austin, Texas, USA). Stage V and VI oocytes were injected with 20 ng cRNA, and currents recorded 1–3 days post-injection.

### 2.3 Electrophysiology

Using two-electrode voltage-clamp, *Xenopus* oocytes were clamped at −60 mV using an OC-725 amplifier (Warner Instruments, Connecticut, USA), Digidata 1322A and the Strathclyde Electrophysiology Software Package (Department of Physiology and Pharmacology, University of Strathclyde, UK). Currents were recorded at 5 kHz and filtered at a frequency of 1 kHz. Micro-electrodes were fabricated from borosilicate glass (GC120TF-10, Harvard Apparatus, Edenbridge, Kent, UK) using a one stage horizontal pull (P-87, Sutter Instrument Company, California, USA) and filled with 3 M KCl. Pipette resistances ranged from 1.0 to 2.0 MΩ. Oocytes were perfused with ND96 at a constant rate of 12 ml min^−1^. Drug application was via a simple gravity fed system calibrated to run at the same rate. Inhibition by test compounds was measured at the GABA EC_50_ (1.6 mM).

Analysis and curve fitting was performed using Prism v4.03 (GraphPad Software, San Diego, California, USA). Concentration–response data for each oocyte were normalised to the maximum current for that oocyte. The mean and S.E.M. for a series of oocytes were plotted against agonist or antagonist concentration and iteratively fitted to the following equation:

(1) $$I_{A}=I_{\min}+\frac{I_{\max}-I_{\min}}{1+10^{n_{H}\left(\log A_{50}-\log A\right)}}$$

where *A* is the concentration of ligand present; *I*_*A*_ is the current in the presence of ligand concentration *A*; *I*_min_ is the current when *A* = 0; *I*_max_ is the current when *A* = ∞, *A*_50_ is the concentration of *A* which evokes a current equal to (*I*_max_ + *I*_min_)/2; and *n*_*H*_ is the Hill coefficient. The relative current amplitudes (*R*_max_) were expressed as the maximal current amplitude evoked by the test compound divided by the maximal current amplitude evoked by GABA.

### 2.4 Docking

Docking was performed using an ELIC crystal structure (pdbid: 2VL0) downloaded from the RCSB Protein Data Bank. A three-dimensional structure of β-endosulfan was extracted from the Cambridge Structural Database (Ref. code: β-Endosulfan = ENSULF). β-Endosulfan was converted into the α conformer and the protonated form constructed in Chem3D Ultra 7.0 and energy-minimized using the MM2 force field.

Docking of the protonated ligand into ELIC was carried out using GOLD 3.0 (The Cambridge Crystallographic Data Centre, Cambridge, UK). The binding site was constrained as a docking sphere with a 20 Å radius surrounding the *C*_α_ of the 6′ residues in chains A and C. These amino acids were chosen based on the binding locations of ligands in eukaryotic Cys-loop receptors, but the docking sphere covered the full length of the transmembrane region of the channel. Ten genetic algorithm runs were performed on each docking exercise using default parameters. The structures were visualised using PyMOL v 1.3 and ViewerLite v 5.0.

## 3 Results

### 3.1 ELIC agonists

**Fig. 2:**
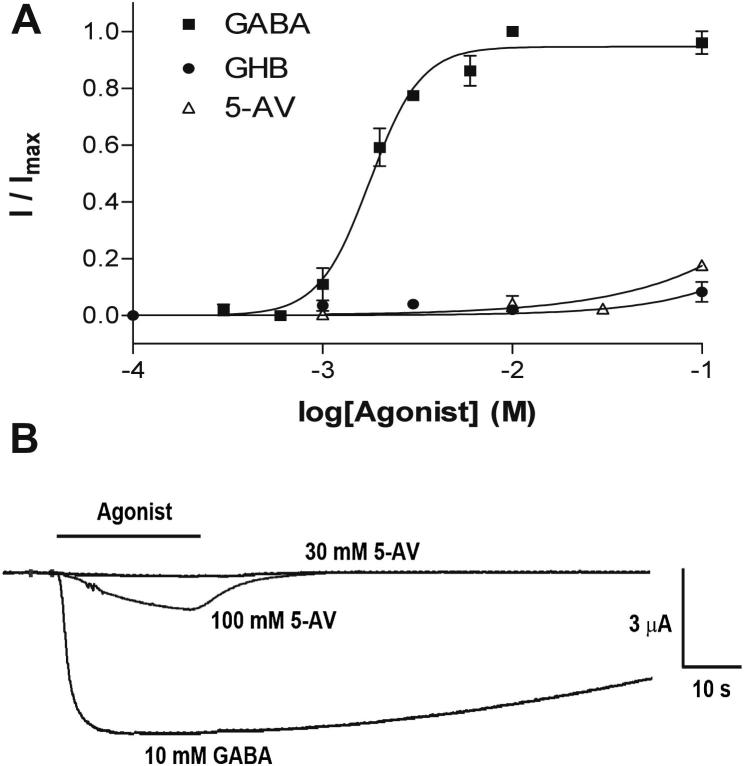
GABA and 5-AV agonist concentration–response curves (A) and example responses (B). The black bar is the application of agonist. Data = mean ± SEM, *n* ≥ 4.

Application of GABA produced large, reversible inward currents (Fig. 2). These will be predominantly Na^+^ currents, given the composition of our buffers and the fact that ELIC is cation-selective (Zimmermann and Dutzler, 2011). Plotting current amplitude against a range of GABA concentrations yielded an EC_50_ of 1.6 mM (pEC_50_ = 2.78 ± 0.04, *n* = 6) and Hill slope of 2.1 ± 0.6. At 1 mM, the amino acid Ala, Arg, Asn, Asp, Cys, Gln, Glu, His, Ile, Leu, Lys, Met, Phe, Pro, Ser, Thr, Trp, Tyr, Val) had no effect on ELIC. At 10 mM several native Cys-loop receptor ligands (ACh, Gly and 5-HT) also yielded no ELIC responses (Table 1).

GABA analogues that activate GABA_A_ receptors were also tested. Gamma-hydroxybutyric acid (GHB) and 5-aminovaleric acid (5-AV) activated ELIC, but required high concentrations (>10 mM) and had small (*R*_max_ < 20%) current amplitudes (Fig. 2), suggesting that they are possibly partial agonists. The GABA analogues muscimol (at 30 μM) α-amino-hydroxybutyric acid, β-alanine, and 3-indole acetic acid (all at 10 mM), and 3-aminopropylphosphonic acid (at 100 mM) had no effect (Table 1).

We also explored a range of compounds that are active in bacterial quorum sensing (White and Finan, 2009). At 10 mM, succinic acid, α-ketoglutarate, α-aminobutyrate, l-glutamate, pyroglutamate, γ-butyrolactone and sodium succinate did not activate ELIC (Table 1).

### 3.2 ELIC antagonists

**Fig. 3:**
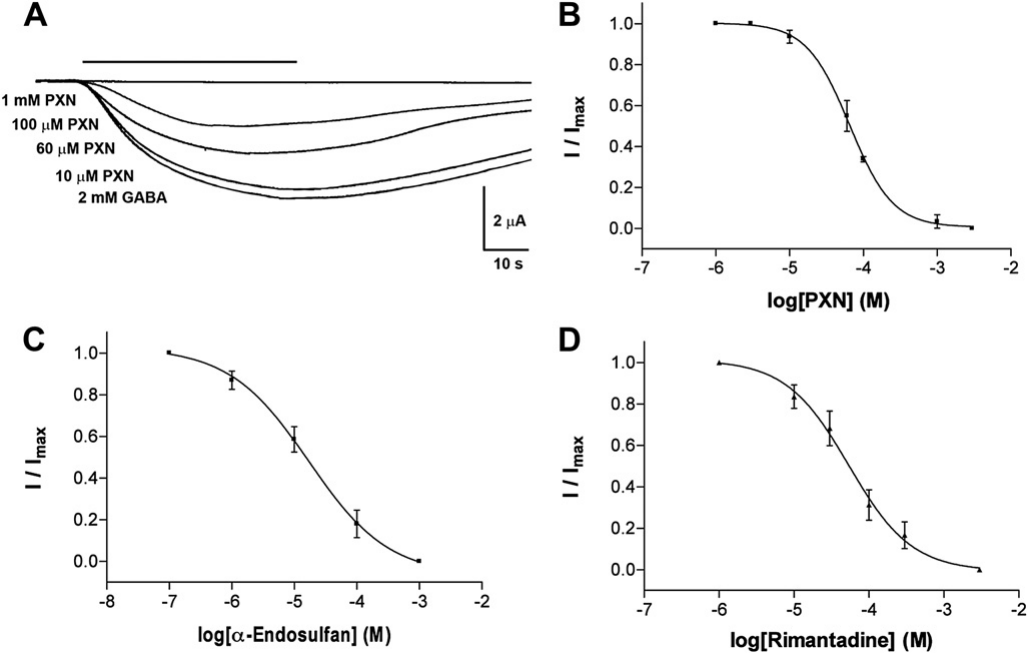
ELIC antagonists. (A) Example traces showing inhibition by picrotoxinin (PXN). Concentration–inhibition curves for PXN (B), α-endosulfan (C), and rimantadine (D). Inhibition was measured at the GABA EC_50_ (1.6 mM). Data = mean ± SEM, *n* ≥ 4. Values derived from the curves can be found in Table 2.

A range of compounds that inhibit or modulate eukaryotic Cys-loop receptors were tested as inhibitors of ELIC (Fig. 3, Table 2). Of the 25 compounds shown in Table 1, 12 inhibited ELIC responses: 2 had IC_50_s < 20 μM, 3 had IC_50_s 20–100 μM, 5 had IC_50_s of 100–1000 μM, and 2 had IC_50_s > 1 mM. Proadifen and α-endosulfan were the most potent, followed by dieldrin, picrotoxinin and rimantadine. A range of amino acids (Pro, His, Gln and Tyr at 1 mM) and the GABA_A_ receptor competitive antagonists bicuculline and gabazine at 100 μM had no effect when co-applied in the presence of GABA. We also tested the quaternary ammonium compounds tetramethylammonium and tetraethylammonium at much higher concentrations, and these compounds inhibited GABA-evoked ELIC responses with IC_50_s close to 20 mM (pEC_50_ = 1.76 ± 0.28 and 1.65 ± 0.06 respectively, *n* = 3). None of the compounds had an effect when applied alone.

We also tested PXN and rimantidine against cysteamine-induced responses as cysteamine is a slightly more efficacious agonist (*R*_max_ = 1. 3 ± 0.1, *n* = 3, cf to GABA; similar to data reported in Zimmermann and Dutzler, 2011). There were no significant differences when compared to inhibition of GABA-induced responses (data not shown).

### 3.3 Ligand docking

**Fig. 4:**
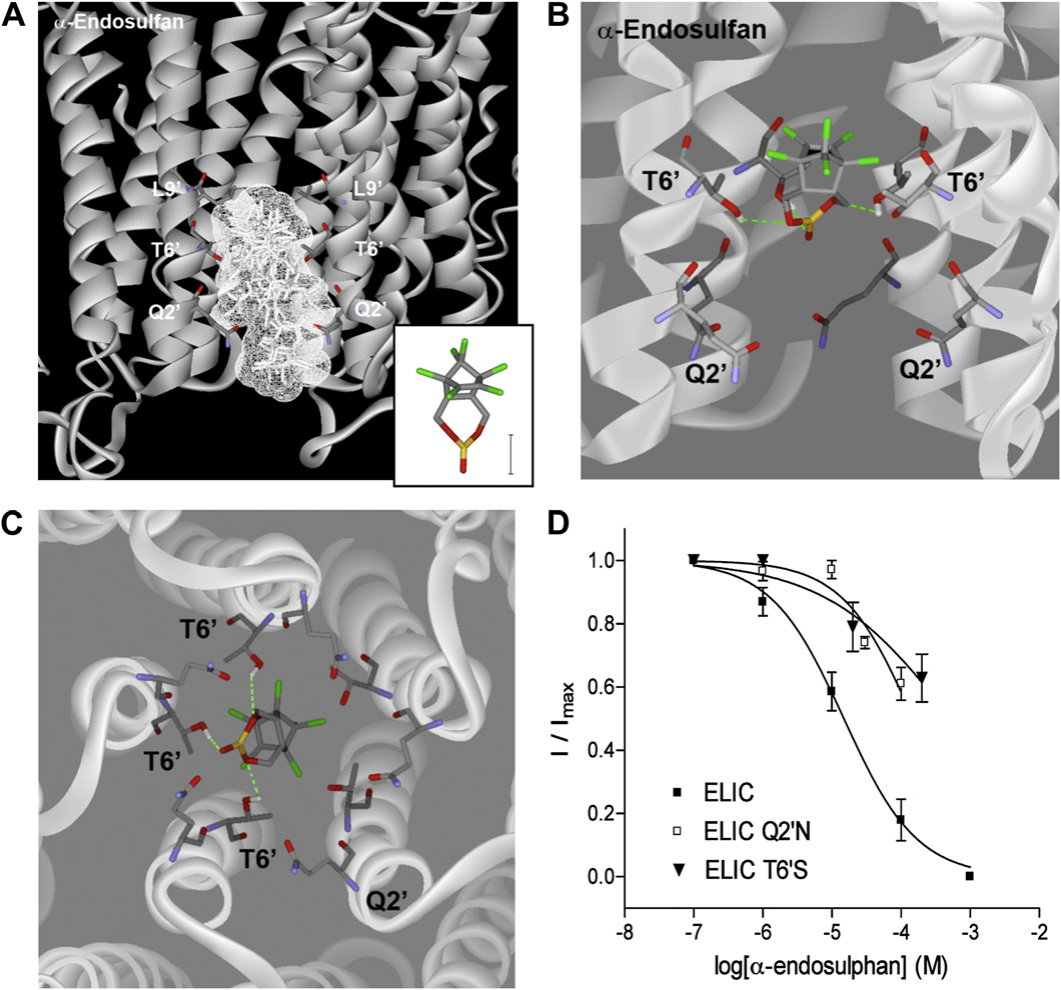
(A) An overlay of all 10 docked poses for α-endosulfan. The channel volume occupied by 10 poses are calculated from the Van der Waals radii and are shown in wireframe. *Inset* Structure of α-endosulfan. Scale = 2.5 Å. (B) A single pose showing the channel from the side. There are hydrogen bond interactions with 6′ Thr residues from adjacent subunits. (C) The same pose is seen from above, looking down towards the cell intererior. (D) Concentration response curves show Q2′N and T6'S mutations caused a decrease in α-endosulfan potency.

To probe possible locations for ligand binding α-endosulfan was docked into the ELIC structure (Fig. 4). It docked close to the 6′ location where it was stabilised by hydrogen bond interactions with Q2′ (2/10 poses) and/or T6′ (6/10) pore-lining residues; in Fig. 4A ten poses are superimposed to show the volume that the docked ligand occupies.

### 3.4 Effects of pore mutations on antagonist potency

To test the predictions of ligand docking, conservative substitutions were made within the ELIC pore at the 2′ and 6′ positions, and the effect on inhibition of the two most potent compounds were examined (Table 3). At both Q2′N and T6'S mutant receptors, the IC_50_ of α-endosulfan was increased >10 fold, supporting a binding location in the pore close to these two residues (Fig. 4D). In contrast, IC_50_s for proadifen were close to wild type, consistent with this compound not being a channel blocking antagonist, as reported for other Cys-loop receptors. At both Q2′N and T6'S mutant receptors GABA EC_50_s and Hill slopes were similar to wild type receptor values.

## 4 Discussion

ELIC is a cationic GABA-gated prokaryotic ligand-gated ion channel that is structurally similar to vertebrate GABA-gated receptors, and, like GABA_A_ receptors, can be modulated by benzodiazepines (Ulens et al., 2011). ELIC can readily be expressed and functionally characterised in *Xenopus* oocytes, but unlike homologous vertebrate receptors, the structure of ELIC at high resolution has been solved (Hilf and Dutzler, 2008). This potentially makes ELIC a good model system for studying structure–function relationships. Here we examine the pharmacology of ELIC. We show that compounds that efficiently activate the receptor are difficult to find, and the novel agonists we identified are of low potency. We also show that classic GABA_A_ competitive antagonists do not inhibit the functional response. However, a range of compounds that act as non-competitive antagonists at GABA_A_ and a range of other Cys-loop receptors also inhibit ELIC responses, suggesting that the pore of ELIC shares some pharmacological similarities to homologous eukaryotic receptors.

It has been previously shown that GABA evokes concentration-dependent responses when ELIC mRNA is injected into *Xenopus* oocytes (Zimmermann and Dutzler, 2011). Our data show similar effects of GABA, and the values obtained from concentration–response curves are comparable. Other compounds that have been previously identified as agonists at ELIC are a range of primary amines, including amino-alcohols and alkyamines (Zimmermann and Dutzler, 2011). New agonists that we identified are 5-AV and GHB, although these are less potent than GABA, and may be partial agonists, as we did not achieve responses > 20% *R*_max_. 5-AV, which is one CH_2_ group longer than GABA, is a low potency partial agonist (EC_50_ = 1.1 mM, *R*_max_ = 0.85) of RDL, a GABA-activated insect receptor (McGonigle and Lummis, 2010). GHB is equivalent to GABA with a hydroxyl group replacing the amino group, and its very low efficacy at ELIC (*R*_max_ < 0.05 at 100 mM) demonstrates the importance of the amino group; this compound has no effect on RDL, supporting a role for the amino group in receptor activation in both classes of GABA-activated receptor (McGonigle and Lummis, 2010). None of the other compounds tested in this study activated ELIC, and the GABA_A_ receptor competitive antagonists were ineffective, suggesting that the ELIC pharmacophore differs significantly from that in the GABA_A_ receptor. Some of the tested compounds are intermediates in quorum sensing, a method of bacterial communication in which ELIC could participate. The absence of effects from these compounds suggests that if ELIC is associated with this mechanism, it is not activated by any of these signalling molecules.

A range of non-competitive antagonists were able to block GABA-evoked responses in ELIC. The majority of these also block GABA-activated Cys-loop receptors, with the most potent (IC_50_ < 20 μM) being α-endosulfan and proadifen, with dieldrin, picrotoxinin (PXN) and rimantidine having IC_50_s < 100 μM. PXN, the more potent component of picrotoxin, blocks a range of Cys-loop receptors including GABA_A_ receptors, while α-endosulfan and dieldrin are cyclodiene insecticides (now rarely used), which block the pore of GABA-activated receptors in both vertebrates and invertebrates (Abalis et al., 1985; Ratra et al., 2001; Chen et al. 2006). Rimantidine and proadifen are not classic channel blockers, although some inhibitory effects have been reported (Spitzmaul et al., 2009; Stouffer et al., 2008). Rimantidine also inhibits GLIC and may act in the pore, although this is unlikely for proadifen, which stabilises the desensitised state. Studies of Cys-loop receptors show interactions with the 2′ and 6′ pore-lining residues contribute to stabilising many channel blocking compounds (e.g. Chiara et al., 2009 Thompson et al., 2011), and more recently, high resolution co-crystal structures have revealed the binding sites of some of these compounds (Hibbs and Gouaux, 2011; Hilf and Dutzler, 2009). Our docking studies indicate that the 2′ and 6′ residues are also important for channel blocking compounds that inhibit ELIC, and our data with α-endosulfan support this hypothesis as mutation of either the 2′ or 6′ residues significantly reduced the potency of this compound.

Compounds that inhibited ELIC responses less potently (IC_50_ > 100 μM) were amantadine, bilobalide, chlorpromazine, fipronil and progesterone. Bilobalide and fipronil block several Cys-loop receptors, including 5-HT_3_, GABA_A_, GluCl, glycine and RDL, by acting at the 6′ residue (Huang et al., 2003; Ratra et al., 2001; Cole et al., 1995; Ikeda et al., 2004; Li and Akk, 2008; Thompson et al., 2011; Islam and Lynch, 2011. Amantadine, chlorpromazine and progesterone also block nACh receptor pores (Buisson and Bertrand, 1998; Chiara et al., 2009; Giraudat et al., 1987, 1989; Matsubayashi et al., 1997; Revah et al., 1990). Quaternary ammonium compounds are open channel blockers of nACh receptors and have been directly observed in co-crystals with GLIC, where they are located close to the 6′ residue (Hilf et al., 2010). Here we show these compounds also block ELIC, albeit at much higher (∼100-fold) concentrations. The low potency of all these channel blocking compounds at ELIC is puzzling, as the pore-lining M2 residues are broadly conserved; we suggest that future studies examine the roles of residues at or close to the entrance to the pore as these may limit access.

**Fig. 5:**
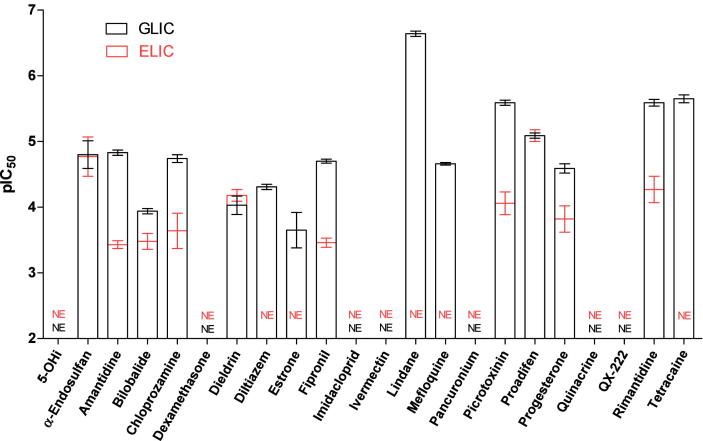
Comparison of pIC_50_ values from ELIC with those previously reported at GLIC (Alqazzaz et al., 2011). Compounds are almost all less potent at ELIC and fewer compounds inhibit responses.

A range of non-competitive antagonists similar to those studied here have also been examined at GLIC (Alqazzaz et al., 2011), but there is limited similarity in the pharmacology of the two receptors (Fig. 5). In general the number of compounds that inhibit ELIC are fewer, and their affinities are lower at ELIC than at GLIC. Only α-endosulfan has a similar IC_50_ at both receptor types (17 μM at both), with 5 other compounds inhibiting both receptors (amantadine, chlorpromazine, fipronil, picrotoxinin, rimantadine) and 5 inhibiting neither (5-hydroxyindole, dexamethasone, imidacloprid, ivermectin, QX-222). This shows that the non-competitive pharmacology of ELIC is less similar to Cys-loop receptors than that of GLIC, and as the majority of ligands studied here are channel blockers in eukaryotes, our data show that the ELIC pore is pharmacologically, as well as structurally, different to those of GLIC, GluCl and the nACh receptor.

In conclusion, we have identified two novel ELIC agonists and a range of compounds that act as antagonists. These are ligands which inhibit a range of Cys-loop receptors (including 5-HT_3_, GABA_A,_ glycine, GluCl and nACh receptors), consistent with the sequence similarities of the M2 regions in all these proteins. These data will be useful when further characterising the mechanism of action of ELIC, but the limited range of ligands that inhibit ELIC, and their lower potencies, indicate that the ELIC pore structure may not be as good as GLIC or other proteins for inferring molecular interactions in the channels of related receptors.

## Conflicts of interest

None.

## Figures and Tables

**Table 1:** Potential ELIC Agonists.

Compound	pEC_50_ (EC_50_)	Hill Slope	*n*
*Cys-loop Agonists*			
1 mM ACh	NE	–	3
1 mM 5-HT	NE	–	3
GABA	2.78 ± 0.04 (1.6 mM)	2.1 ± 0.6	6
1 mM Glycine	NE	–	3
*GABA Analogues*			
30 μM muscimol	NE	–	3
10 mM 3-indole acetic acid	NE	–	5
100 mM 3-aminophosphonic acid	NE	–	5
10 mM β-alanine	NE	–	3
5-aminovaleric acid^a^	17 ± 1% of *R*_max_ at 100 mM	–	3
γ-hydroxybutyric acid^a^	8.2 ± 3% of *R*_max_ at 100 mM	–	3
*Quorum Sensing*			
10 mM succinic acid	NE	–	3
10 mM α-ketoglutarate	NE	–	3
10 mM α-aminobutyrate	NE	–	3
10 mM l-glutamate	NE	–	3
10 mM pyroglutamate	NE	–	3
10 mM γ-butyrolactone	NE	–	3
10 mM sodium succinate	NE	–	3
10 mM α-amino hydroxbutyric acid	NE	–	3
*Amino Acids*			
10 mM Alanine	NE	–	4
10 mM Arginine	NE	–	4
10 mM Asparagine	NE	–	3
10 mM Aspartate	NE	–	3
10 mM Cysteine	NE	–	6
10 mM Glutamine	NE	–	3
10 mM Glycine	NE	–	4
10 mM Histidine	NE	–	4
10 mM Isoleucine	NE	–	4
10 mM Leucine	NE	–	3
10 mM Lysine	NE	–	4
10 mM Methionine	NE	–	4
10 mM Phenylalanine	NE	–	3
10 mM Proline	NE	–	3
10 mM Serine	NE	–	4
10 mM Threonine	NE	–	4
10 mM Tryptophan	NE	–	4
10 mM Tyrosine	NE	–	4
10 mM Valine	NE	–	4

NE = No Effect at the concentration shown. *R*_max_ = maximal current relative to 10mM GABA.

a For 5-AV and GHB, EC_50_ values could not be calculated as the agonist responses did not saturate.

**Table 2:** Potential ELIC antagonists.

Ligand	Known LGIC Targets	pIC_50_ (Mean ± SEM)	IC_50_ (μM)	*n*_*H*_	*n*
5-hydroxyindole	5-HT_3_, α7 nACh, GABA_A_	NI	–	–	4
α-endosulfan	GABA_A_, Gly	4.77 ± 0.15	17	0.7 ± 0.2	4
Amantadine	nACh	3.43 ± 0.03	370	2.5 ± 0.4	4
Bicuculline	GABA_A_	NI*	–	–	3
Bilobalide	5-HT_3_, GABA_A_, Gly	3.48 ± 0.06	330	1.3 ± 0.2	4
Chlorpromazine	5-HT_3_, nACh	3.64 ± 0.12	230	1.2 ± 0.4	5
Dexamethasone	5-HT_3_	NI	–	–	4
Dieldrin	GABA_A_, Gly	4.18 ± 0.14	66	1.1 ± 0.1	4
Diltiazem	5-HT_3_, α7 nACh, GABA_A_	NI	–	–	4
Estrone	5-HT_3_	NI	–	–	5
Fipronil	GluCl, GABA_A_, Gly	3.46 ± 0.04	350	1.8 ± 0.4	3
Gabazine (SR-95531)	GABA_A_	NI*	–	–	5
Imidaclopride	nACh	NI	–	–	4
Ivermectin	GluCl, Gly	NI	–	–	3
Lindane	GABA_A_, Gly	NI	–	–	4
Mefloquine	5-HT_3_, nACh	NI	–	–	3
Pancuronium	nACh	NI	–	–	3
Picrotoxinin	5-HT_3,_ GABA_A_, Gly	4.06 ± 0.10	96	1.2 ± 0.6	3
Proadifen	nACh	5.09 ± 0.04	8.1	2.9 ± 0.6	4
Progesterone	5-HT_3_	3.82 ± 0.09	150	1.8 ± 0.58	4
Quinacrine	nACh	NI	–	–	3
QX-222	nACh	NI	–	–	4
Rimantadine	nACh	4.27 ± 0.03	54	1.0 ± 0.2	4
Tetracaine	5-HT_3_, nACh	NI	–	–	5

NI = no inhibition at 10 mM, NI* = no inhibition at 100 μM.

**Table 3:** Antagonist properties at M2 mutant receptors.

	GABA pEC_50_ (EC_50_)	α-endosulfan pIC_50_ (IC_50_)	Proadifen pIC_50_ (IC_50_)
Wild Type	2.78 ± 0.04 (1.6 mM)	4.77 ± 0.15 (17 μM)	5.09 ± 0.04 (8.1 μM)
Q2′N	2.74 ± 0.02 (1.8 mM)	NI (>100 μM)	5.54 ± 0.09 (2.9 μM)
T6'S	2.75 ± 0.02 (1.8 mM)	NI (>100 μM)	5.29 ± 0.04 (5.1 μM)

NI = IC_50_ was not reached at the highest concentration tested (10^−4^ M). See Fig. 4D.
